# A systematic review of the quality of conduct and reporting of systematic reviews and meta-analyses in paediatric surgery

**DOI:** 10.1371/journal.pone.0175213

**Published:** 2017-04-06

**Authors:** Paul Stephen Cullis, Katrin Gudlaugsdottir, James Andrews

**Affiliations:** 1 Department of Surgical Paediatrics, Royal Hospital for Children, Glasgow, United Kingdom; 2 School of Medicine, University of Glasgow, Glasgow, United Kingdom; Cardiff University, UNITED KINGDOM

## Abstract

**Objective:**

Our objective was to evaluate quality of conduct and reporting of published systematic reviews and meta-analyses in paediatric surgery. We also aimed to identify characteristics predictive of review quality.

**Background:**

Systematic reviews summarise evidence by combining sources, but are potentially prone to bias. To counter this, the Preferred Reporting Items for Systematic Reviews and Meta-Analyses (PRISMA) was published to aid in reporting. Similarly, the Assessing the Methodological Quality of Systematic Reviews (AMSTAR) measurement tool was designed to appraise methodology. The paediatric surgical literature has seen an increasing number of reviews over the past decade, but quality has not been evaluated.

**Methods:**

Adhering to PRISMA guidelines, we performed a systematic review with *a priori* design to identify systematic reviews and meta-analyses of interventions in paediatric surgery. From 01/2010 to 06/2016, we searched: MEDLINE, EMBASE, Cochrane, Centre for Reviews and Dissemination, Web of Science, Google Scholar, reference lists and journals. Two reviewers independently selected studies and extracted data. We assessed conduct and reporting using AMSTAR and PRISMA. Scores were calculated as the sum of reported items. We also extracted author, journal and article characteristics, and used them in exploratory analysis to determine which variables predict quality.

**Results:**

112 articles fulfilled eligibility criteria (53 systematic reviews; 59 meta-analyses). Overall, 68% AMSTAR and 56.8% PRISMA items were reported adequately. Poorest scores were identified with regards *a priori* design, inclusion of structured summaries, including the grey literature, citing excluded articles and evaluating bias. 13 reviews were pre-registered and 6 in PRISMA-endorsing journals. The following predicted quality in univariate analysis:, word count, Cochrane review, journal h-index, impact factor, journal endorses PRISMA, PRISMA adherence suggested in author guidance, article mentions PRISMA, review includes comparison of interventions and review registration. The latter three variables were significant in multivariate regression.

**Conclusions:**

There are gaps in the conduct and reporting of systematic reviews in paediatric surgery. More endorsement by journals of the PRISMA guideline may improve review quality, and the dissemination of reliable evidence to paediatric clinicians.

## Background

Systematic reviews and meta-analyses have an increasingly important role in modern healthcare. They are used to appraise evidence, inform policy, construct guidelines and assess cost-effectiveness of interventions. However, both systematic reviews and meta-analyses can potentially be biased through the selection, analysis and reporting of included studies. In recent years, attempts have been made to encourage authors to report reviews following an agreed protocol and in doing so improve the conduct of reporting of such reviews. The Preferred Reporting Items for Systematic Reviews and Meta-Analyses (PRISMA) statement evolved from the earlier Quality of Reporting of Meta-analyses (QUORUM) collaboration checklist, both of which had been designed to form a framework of reporting for authors of systematic reviews and meta-analyses [1]. Since it’s publication in 2009, PRISMA has been endorsed by many major healthcare journals, many more recommend adherence and its popularity is growing. Several extensions followed publication of PRISMA and there are still more developments underway, including tools focusing on the paediatric population. Whilst PRISMA encourages quality reporting of systematic reviews, the Assessing the Methodological Quality of Systematic Reviews (AMSTAR) measurement tool was designed to appraise systematic review methodology critically. It has since been validated and proven popular as a simple means of assessing the quality of reviews [2–3].

Research in surgery presents unique challenges in producing high quality evidence comparing interventions, but this is particularly true in the surgery of childhood. Ethical approval for research can be challenging in paediatrics, not least because of issues with consent [4]. Furthermore, recruitment is often challenging and the incidence of many paediatric conditions is low, which hinders the ability to power studies appropriately, especially when the outcome measure is itself uncommon. Examples of trials in paediatrics hindered by issues with study recruitment, include the VICI [5] and PLUTO [6] trials, and multicenter randomised-controlled trials comparing laparotomy with drainage for neonatal perforation [7–8]. Potentially as a consequence of such difficulties, retrospective case series account for almost half of the paediatric surgical literature. Despite their suitability, multicentre trials are uncommon [9]. Therefore, cumulative tools have become useful adjuncts in the paediatric surgical literature to draw conclusions on a multitude of smaller studies [10–11].

Our primary aim was to evaluate the quality of conduct and reporting of published systematic reviews and meta-analyses in paediatric surgery, including general surgery of childhood, neonatal surgery and paediatric urology. Our secondary aim was to identify any article, author or journal characteristics associated with high quality reviews.

## Methods

We employed a methodology not dissimilar to Adie et al. (which did not focus on the paediatric surgical literature, but instead, the quality of reporting and methodology of systematic reviews and meta-analyses in the surgical literature in general [12] and McGee at al. (which focused on systematic reviews and meta-analyses of randomised controlled trials of any surgical interventions in children) [13].

### Registration and protocol

Registration of the review with PROSPERO, an international prospective register of systematic reviews, was attempted, however, purely methodological reviews are not included in the database. The *a priori* review protocol may therefore be sought from: https://drive.google.com/open?id=0B49a9IgOcHHRbWlKYnRfR1ZYTjA. This systematic review was reported in accordance with the PRISMA statement^1^.

### Search strategy

**Fig 1:**
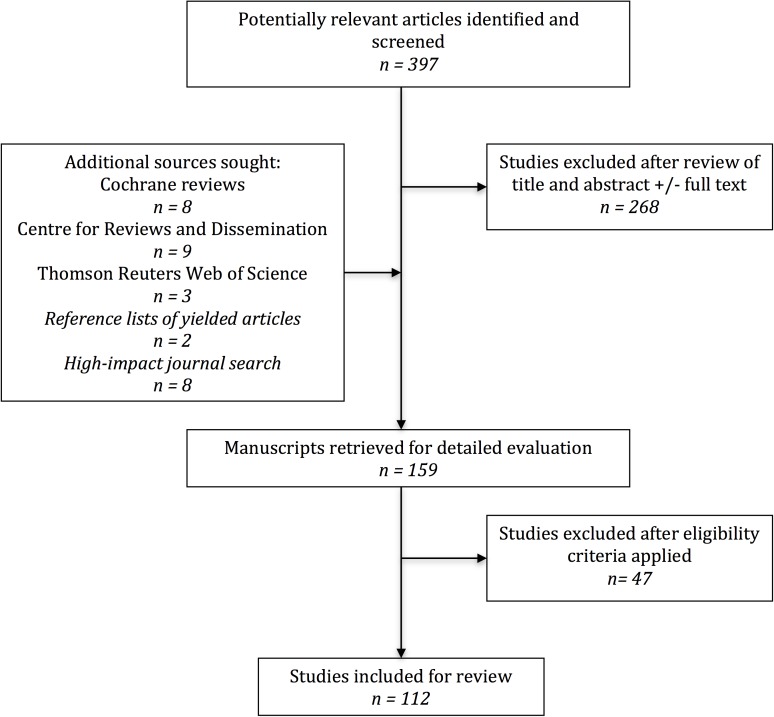
PRISMA flow diagram.

A systematic search of the English literature was performed on 10th June 2016 to identify systematic reviews and meta-analyses focusing on paediatric surgical interventions published from 1st January 2010 to 10th June 2016. The former date was selected because the original PRISMA statement was published and disseminated in multiple medical and surgical journals in mid-2009. An initial electronic search was conducted using MEDLINE and EMBASE databases. The search strategy is shown in S1 Table and the PRISMA flow diagram in Fig 1. A similar search was performed of the Cochrane Database of Systematic Reviews (by searching all articles manually within the period studied under topics: Cancer, Child Health, Endocrine & Metabolic, Gastroenterology & Hepatology, Kidney Disease, Methodology, Neonatal Care, Pregnancy & Childbirth, Urology and Wounds), Centre for Reviews and Dissemination database (similar to the search conducted in S1 Table), Thomson Reuters Web of Science (similar to the search conducted in S1 Table), and Google Scholar (searching for articles with “surgery”, “intervention” or “procedure” in the title and including either “paediatric”, “pediatric”, “neonatal”, “neonate”, “infant”, “child”, “children”, “adolescent” or “toddler”). The reference lists of included articles were also searched, in addition to hand-searching of various relevant high-impact journals (S2 Table).

### Eligibility criteria

Inclusion and exclusion criteria are highlighted in Table 1. The titles and abstracts of the retrieved articles were screened independently by two authors (P.S.C. and K.G.) using the inclusion criteria, and the full texts of yielded articles were subsequently sought. Eligibility criteria were then applied to the retrieved set of articles by the same authors. Disputes were presented to the third author (J.A.) and a consensus was reached. It should be noted that we took the definition of paediatric surgical interventions to include any performed commonly by a paediatric surgeon in the UK. Normally this role combines general surgery of childhood, paediatric urology and neonatal surgery only, as defined in the UK Joint Committee on Surgical Training’s Certificate of Completion of Training documentation [14].

**Table 1:** Eligibility criteria employed.

Inclusion criteria	Exclusion criteria
Study identified as a systematic review, with or without meta-analysis, data synthesis or quantitative overview	Studies focusing on other paediatric surgical specialties, foetal medicine or paediatric anaesthesia
English language	Grey literature (i.e. manuscripts not published in peer-review journals or books)
Published from 1st January 2010 to 10th June 2016 (online or in print)	Majority (>50%) patients within included studies of review adult (>18 years of age) and/or paediatric patient data not analysed separately
Full text published article	Non-human subjects
Studies focusing on intervention(s) during childhood within field of the general surgery of childhood or paediatric or urology, to include neonatal surgery	

### Data extraction

An electronic data collection form was developed by two authors (P.S.C. and K.G.). Data extraction was then performed independently, with interobserver reliability assessed using the kappa statistic. General characteristics of systematic reviews were extracted, including details of authors (number, gender, department, country(ies) of origin), the study (systematic review or meta-analysis, type of comparison, number of studies included, funding sources), the journal (name, type, impact factor, h5 index, PRISMA endorsement, PRISMA adherence suggested in author guidelines) and the article (word count, registration, PRISMA adherence described). These were selected as descriptive comparators, however, most of these variables have been hypothesised as being associated with quality, and we used them in the exploratory analyses described later [12].

### Quality appraisal

Quality of studies included was assessed by two means. The AMSTAR checklist was designed to evaluate systematic reviews and guide prospective review conduct. It consists of an 11-item tool that we employed to score texts. A single point was given for each item if reporting was considered adequate, no points if inadequate, and not applicable if that item was not relevant to the text, for example, combining data in quantitative synthesis or assessing publication bias in the context of a systematic review without a meta-analysis [2]. Therefore, the maximum achievable score was 11. Secondly, we used the PRISMA checklist in a similar fashion, achieving a maximum score of 27 for texts [1]. Since for several items, such as those relating to meta-analysis in the context of a systematic review, scores were not applicable, AMSTAR and PRISMA items were to be reported as global percentages of applicable items. It is important to note that AMSTAR scores relate to methodological quality whilst PRISMA relates to reporting quality.

### Sample size calculation

Sample size calculation was not performed as all systematic reviews published during the search period and meeting the eligibility criteria were to be included. The number of articles included would influence univariate and multivariate regression analyses. We did not limit the number of exploratory variables in regression analysis, however, because regression analysis was a secondary objective and because the journal, author, study and article characteristics were defined before statistical analysis.

### Data analysis

A biostatistician was consulted for assistance with statistical analysis. Simple descriptive analysis was performed for variables relating to author, study, journal and article characteristics (see Data Extraction section). The general characteristics of systematic reviews extracted were used as exploratory variables of AMSTAR and PRISMA scores, separately. Namely, we included: number of authors, medical/surgical versus university department of first author, Anglophonic versus other country of origin of first author, review compares treatment versus no comparison, number of studies included, whether the study was funded, whether it was a Cochrane review or not, journal impact factor, journal h5 index, whether the journal endorses PRISMA, whether the journal suggests PRISMA adherence in author guidelines, article word count, whether the review was registered, and whether PRISMA adherence was reported. In univariate and multivariate modelling, a p value <0.05 was considered statistically significant. Univariate linear regression was first performed for each variable, and subsequently, those variables with a p value <0.1 were combined in stepwise backward multiple regression analysis. Those significant variables in each multiple regression analysis were combined in the final multiple regression model. The above analyses were performed on Minitab statistical software (release 16; Minitab, Minitab Inc, State College, Philadelphia).

## Results

### Search results

112 articles yielded met formal eligibility criteria and were included for analysis, comprising 53 systematic reviews which did not contain a meta-analysis, and 59 systematic reviews with meta-analyses. The PRISMA flow diagram is illustrated in Fig 1 and excluded studies and reasoning for exclusion are listed in Table 2 below [15–60].

**Table 2:** Excluded studies and reasoning.

Reason for exclusion	Articles excluded (reference number)
Not regarding specific paediatric surgical or urological interventions	16–25, 27–35, 37, 38, 41–52, 54–60
Majority (>50%) patients within included studies of review adult (>18 years of age) and/or paediatric patient data not analysed separately	15,36
Not a full text original manuscript	26, 39, 40, 53

### General characteristics

The characteristics of studies included in the final analysis [61–172] are listed in Table 3. The mean number of authors per article was 5; 63.4% were affiliated with a department of paediatric surgery. Articles were published by 101 first authors, from a total of 22 countries. The UK was responsible for more publications than any other country (25.9%), followed by Canada (13.4%), China (13.4%) and the USA (10.7%). The majority (57.1%) of yielded articles were by first authors of anglophonic countries whilst 13.4% articles represented international collaborations.

**Table 3:** Characteristics of included studies.

Characteristic			n
Authors	Number of authors (%)	1–3	40 (35.7)
		4–6	49 (43.8)
		>6	23 (20.5)
	Department of first author (%)	Paediatric surgery or urology	71 (63.4)
		Other surgical subspecialty	18 (16.1)
		Research/university/epidemiology	17 (15.2)
	Gender of first author (%)	Male	73 (65.2)
		Female	39 (34.8)
	Country of first author (%)	UK	29 (25.9)
		Canada	15 (13.4)
		China	15 (13.4)
		USA	12 (10.7)
		Germany	7 (6.3)
		Netherlands	7 (6.3)
	First author from Anglophonic country (%)		64 (57.1)
	International collaborative authorship (%)		15 (13.4)
Journal	Type of journal (%)	Paediatric surgery or urology	69 (61.6)
		Other surgical subspecialty	19 (17)
		Surgery, in general	7 (6.3)
		Medicine, in general	6 (5.4)
		Cochrane	5 (4.5)
		Paediatrics	5 (4.5)
	Journal title (%)	Journal of Pediatric Surgery	27 (24.1)
		Pediatric Surgery International	20 (17.9)
		European Journal of Pediatric Surgery	14 (12.5)
		Journal of Pediatric Urology	5 (4.5)
		Cochrane	5 (4.5)
	h5 index (median with IQR, and range)		31.5 (11.3, 8–161)
	Impact factor (median with IQR, and range)		1.4 (0.9, 0–8.3)
	PRISMA-endorsing journal (%)		6 (5.4)
	PRISMA adherence advised by journal (%)		13 (11.6)
Article	Review theme (%)	Generic or emergency	32 (28.6)
		Gastrointestinal (upper or lower)	38 (33.9)
		Urology	26 (23.2)
		Thoracic	12 (10.7)
		Oncology	4 (3.6)
	Type of comparison (%)	Surgery vs surgery	70 (62.5)
		Non-surgery vs surgery	12 (10.7)
		No comparison	30 (26.8)
	Pre-registered (%)		13 (11.6)
	Funding (%)		16 (14.3)
	PRISMA adherence stated within article (%)		30 (26.8)
	Number of studies included (median with IQR, and range)		13 (17, 0–98)
	Word count (median with IQR, and range)		5798 (3028, 2000–47914)

Articles were published in 31 different journals with the majority from journals dedicated to paediatric surgery or urology (61.6%). Median h5 index was 31.5 whilst median impact factor was 1.4. The most popular three journals were: the Journal of Pediatric Surgery (24.1%), Pediatric Surgery International (17.9%) and the European Journal of Paediatric Surgery (12.5%). The top three journals (with more than one publication yielded) as rated by highest mean AMSTAR score achieved were: Cochrane Database of Systematic Reviews (93%), Annals of Surgery (55%) and the Journal of Urology (47%). For PRISMA scores, the respective top three journals were: Cochrane Database of Systematic Reviews (93%), Annals of Surgery (87%) and the Journal of Gastrointestinal Surgery (83%). Only 5.4% articles were published in PRISMA-endorsing journals whilst only 11.6% were published in journals which encourage PRISMA adherence.

More than one third of reviews were on the subject of gastrointestinal surgery, and two-thirds compared surgical interventions. Only 11.6% reviews were pre-registered. Median journal impact factor was 1.4 (IQR 0.9) and median h5 index was 31.5 (IQR 11.3).

### AMSTAR and PRISMA scores

**Fig 2:**
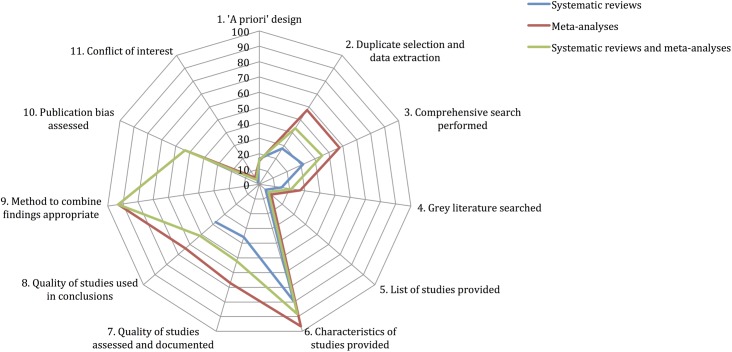
star chart illustrating AMSTAR scores achieved for systematic reviews, meta-analyses and their cumulative total, as percentage of adequately reported items.

**Fig 3:**
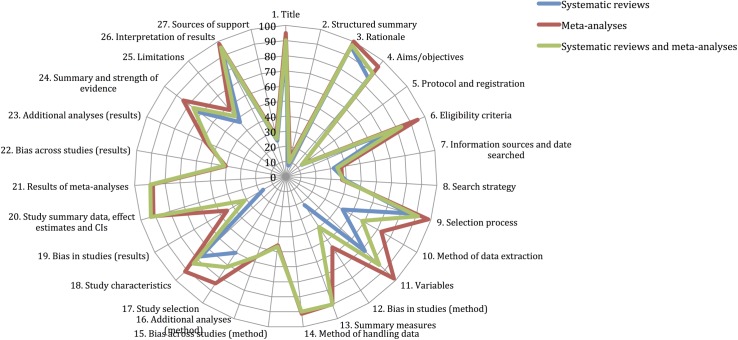
star chart illustrating PRISMA scores achieved for systematic reviews, meta-analyses and their cumulative total, as percentage of adequately reported items.

Figs 2 and 3 illustrate the proportion of systematic reviews, meta-analyses and both systematic reviews and meta-analyses that adequately reported each AMSTAR and PRISMA item. Overall, 68% AMSTAR and 56.8% PRISMA items were described adequately. AMSTAR items reported well were: 6. Characteristics of studies provided (88.3%) and 9. Methods to combine findings appropriate (93.1%). AMSTAR items which scored particularly poorly were: 1. *A priori* design (15.9%), 4. Grey literature searched (21.2%), 5. List of studies provided (8%), and 11. Conflict of interest inclusion (3.5%). Conversely, PRISMA items reported well were: 1. Title (90.3%), 3. Rationale (97.3%), 4. Aims/objectives (89.4%), 6. Eligibility criteria (83.2%), 9. Selection process (91.2%), 11. Variables (85%), 18. Study characteristics (83.8%) and 26. Interpretation of results (95.6%). PRISMA items which scored particularly poorly were: 2. Structured summary (9.7%), 5. Protocol and registration (13.3%), 7. Information sources and date searched (34.5%), 12. Bias in studies with regards methods (39.8%), 15. Bias across studies with regards methods (46.7%), 19. Bias in studies with regards results (31.5%), 22. Bias across studies with regards results (41.4%), and 27. Sources of support (25.7%). meta-analyses achieved notably higher scores for each AMSTAR and PRISMA item, except for AMSTAR item 1. *A priori* design and PRISMA item 8. Search strategy.

### Interobserver reliability

The overall kappa statistic for AMSTAR and PRISMA items was 0.89, equating to almost perfect agreement. For no items was agreement less than substantial. Three items were rated <0.7: (a) AMSTAR item 2. Duplicate study selection and data extraction, (b) AMSTAR item 3. Comprehensive literature search, and (c) PRISMA item 8. Full electronic search strategy. For AMSTAR item 2 and PRISMA item 8, the wording of manuscripts was often unclear such that deciding on whether these criteria were fulfilled was challenging. For AMSTAR item 3, there was some initial uncertainty as to whether or not searching the reference lists of retrieved articles counted as a supplementary strategy in its own right.

### Statistical analyses

Linear regression of exploratory variables using AMSTAR and PRISMA separately as dependent variables identified several significant trends displayed in Tables 4 and 5. The following factors were significant in univariate linear regression with regards AMSTAR score: first author affiliation with research institute or university, review includes a comparison of interventions, article word count, article is a Cochrane review, journal h-index, journal impact factor, journal endorses PRISMA, journal suggests PRISMA adherence in the author guidance, and review registration. In its respective multiple regression analysis, the following variables were significant: first author affiliation with research institute or university and review registration. The following factors were significant in univariate linear regression with regards PRISMA score: review includes a comparison of interventions, article word count, article is a Cochrane review, journal h-index, journal impact factor, journal endorses PRISMA, journal suggests PRISMA adherence in the author guidance, review article mentions PRISMA adherence, and the review registration. In its respective multiple regression analysis, the following variables were significant: review includes a comparison of intervention and review article mentions PRISMA adherence.

**Table 4:** showing only those variables that were identified as significant (p < 0.05) in univariate regression for AMSTAR scores and the results of subsequent input to multiple regression, again showing only significant results. The overall model fit for final multiple regression equation was R^2^ = 0.51. Change in AMSTAR score refers to the expected change in AMSTAR score with a unit increase in the variable assessed with all other variables being constant. N.B. systematic review (SR); meta-analysis (MA); confidence interval (CI).

	UNIVARIATE REGRESSION	MULTIVARIATE REGRESSION
Exploratory variable	Expected change in AMSTAR score (as % change with 95% CI)	Expected change in AMSTAR score (as % change with 95% CI)
First author affiliated with research institute/university versus no affiliation	+13 (0.2 to 25.7)	
SR or MA compares treatment versus no comparison	+29.6 (20.6 to 38.5)	+25.3 (18 to 32.6)
Article word count (per 1000 words)	+1.7 (1 to 2.5)	
Cochrane review versus non-Cochrane	+55.1 (35.1 to 75.1)	
Journal h-index	+0.4 (0.3 to 0.6)	
Journal impact factor	+6.3 (3.9 to 8.7)	
Journal endorses PRISMA versus no endorsement	+50.5 (32.2 to 68.9)	
Journal suggests PRISMA adherence versus does not	+32.5 (19.4 to 45.7)	
Review registered versus not	+43 (31.3 to 55.3)	+37.8 (27.6 to 48)

**Table 5:** showing only those variables that were identified as significant (p < 0.05) in univariate regression for PRISMA scores and the results of subsequent input to multiple regression, again showing only significant results. The overall model fit for final multiple regression equation was R^2^ = 0.29. N.B. Change in PRISMA score refers to the expected change in PRISMA score with a unit increase in the variable assessed with all other variables being constant. NB. systematic review (SR); meta-analysis (MA); confidence interval (CI).

	UNIVARIATE REGRESSION	MULTIVARIATE REGRESSION
Exploratory variable	Expected change in PRISMA score (as % change with 95% CI)	Expected change in PRISMA score (as % change with 95% CI)
SR or MA compares treatment versus no comparison	+21.5 (13.8 to 29.2)	+19.6 (12.3 to 26.9)
Article word count (per 1000 words)	+1.2 (0.6 to 1.8)	
Cochrane review versus non-Cochrane	+31.7 (14 to 49.3)	
Journal h-index	+0.3 (0.2 to 0.4)	
Journal impact factor	+4.9 (2.9 to 6.8)	
Journal endorses PRISMA versus no endorsement	+29.6 (13.5 to 45.8)	
Journal suggests PRISMA adherence versus does not	+23.3 (12.1 to 34.5)	
Article mentions PRISMA versus does not	+16.3 (8.1 to 24.4)	+13.6 (6.3 to 20.8)
Review registered versus not	+26.2 (15.2 to 37.1)	

## Discussion

### Findings in context

This review has evaluated the adequacy of systematic reviews and meta-analyses in the published paediatric surgical literature, and has highlighted areas of particular concern with regards the conduct and methodology of such reviews. Overall, compliance with the AMSTAR checklist was moderate, with two thirds (68%) of AMSTAR items reported adequately amongst all reviews. Similarly, compliance with the PRISMA guidelines was poorer with approximately half (56.8%) of PRISMA items reported adequately. Globally poor scores were identified with regards *a priori* design, review registration, inclusion of structured summaries, including the grey literature, citing excluded articles, evaluating bias and inclusion of conflict of interest statements.

Overall, meta-analyses score higher with regards AMSTAR scores and PRISMA compliance, than systematic reviews alone. AMSTAR score was positively associated with the review registration and first author affiliation with a research institute or university, whilst compliance with PRISMA was positively associated with the review article mentioning PRISMA adherence and including a comparison of surgical interventions (the latter variable may be explained however by the increased likelihood that meta-analyses compared interventions). No other review characteristics were significant in the final multivariate regression analyses.

The Oxford level of evidence grading system highlights that cumulative evidence obtained from several studies combined is of higher quality than their individual research study components, reflected in the fact that systematic reviews are a step above their constituent studies [10]. It is therefore an easy and often incorrect assumption that systematic reviews and meta-analyses equate to quality evidence. The GRADE system, however, places less strength on systematic reviews and meta-analyses but still considers such cumulative analyses of RCTs the highest possible form of evidence alongside individual RCTs [11]. The methodology and reporting of systematic reviews and meta-analyses are prone to flaws as much as any other form of medical research, and the Oxford grading system does make this clear. We have highlighted that paediatric surgery is no different with this regard.

Only two reviews achieved perfect scores with regards the AMSTAR criteria [122,157]; no articles were considered perfect in relation to their PRISMA score. We, the authors, are no less guilty of failing to report all items on the PRISMA checklist to their entirety in the past [80,173]; with the current study, best attempts were made to follow the checklist. It is paramount that investigators planning systematic reviews and meta-analyses adhere to PRISMA guidance, to ensure methodological robustness and, by improving quality of reporting, optimise the communication of the review and findings to its readers. In turn, this should help clinicians keep up-to-date with the current evidence, and subsequently, improve the care of children affected by surgical conditions.

The issue of reporting in paediatric surgery is not limited to systematic reviews and meta-analyses. Randomised controlled trials remain rare, accounting for <0.05% of all publications in the field of paediatric surgery [174]. Similar to the PRISMA statement, the Consolidated Standards of Reporting Trials (CONSORT) guideline was designed to improve reporting of trials by means of a standardised, evidence-based checklist [175]. Despite its first publication in 1996, trials in paediatric surgical specialties are poorly reported, with only 2% of trials meeting the full CONSORT criteria [176]. Recently, paediatric surgical guidelines have been scrutinised in a similar manner. Shaywer et al. used the Appraisal of Guidelines for Research and Evaluation Instrument to assess the quality of guidelines published in major paediatric surgical journals. Whilst specific areas achieved moderate scores, overall quality was considered poor and they highlighted that important aspects of guidelines are still underreported [177].

*A priori* study design was adequately reported in only 16% of studies. To explore whether or not this was a reporting or methodological issue, we searched the Centre for Reviews and Dissemination database to identify registered reviews. This confirmed that this low figure relates to failings to register reviews rather than failure to report registration, with PROSPERO, at least. We did not identify a single article that was registered yet did not document this amongst its text. Having a pre-determined protocol is important because it may restrict the opportunities for biased *post hoc* changes in methodology [178]. Our data suggests a positive association between review registration and quality. We were unable to identify any other such association in the literature with regards systematic reviews, however, there is evidence that registration is positively associated with better reporting of clinical trials [179]. Inclusion of the grey literature was considered adequate for 21% of included studies. This is another important aspect of reviews to minimise publication bias. 8% studies achieved adequate scores for providing lists of studies. To achieve this, the AMSTAR checklist is clear that a list of both included *and* excluded studies must be provided [2]. Almost all studies provided the former citations, yet only 9 provided the latter, most of which were Cochrane reviews. Similarly, only 3.5% studies were considered adequate in relation to conflict of interest statements. The AMSTAR checklist insists that both the sources of support or funding for the review itself *and* the included studies must be reported^2^. Again it is the latter aspect that is, in general, poorly reported. This is reflected in the fact PRISMA item 27 Funding was adequately reported in 26% studies, an item which we considered adequate if only the review funding was listed as worded in the PRISMA checklist.

McGee et al. have evaluated the quality of conduct and reporting of systematic reviews of RCTs of surgical procedures in children^13^. This was not limited to paediatric surgery and urology, but instead all surgical subspecialty publications were included, and publications until the end of 2010 were assessed, largely before publication of PRISMA. Despite the broad nature of reviews and lengthy timescale assessed, only 15 systematic reviews were included in the final analysis, compared with 112 in our study. This difference likely reflects the paucity of RCTs in surgical subspecialties of childhood and the snowballing popularity of systematic reviews and meta-analyses in the surgical literature. Similar to the current study, McGee et al. found that PRISMA items 15 and 22, relating to the risk of bias across studies with regards their methods and results, achieved some of the lowest PRISMA scores. An important difference between our study and theirs is the proportion of included studies from the Cochrane Collaboration. Almost 90% of their systematic reviews were from this database, as opposed to <5% in the current study. This fact reflects many other differences in PRISMA scores achieved. They found that PRISMA item 1 was poorly reported i.e. the inclusion of systematic review or meta-analysis in the review title. Nevertheless, the Cochrane Collaboration tends not to include either “systematic review” or “meta-analysis” within the title, perhaps because inclusion in the database implies its systematic review methodology. On the contrary, McGee et al. found PRISMA items for registration, structured summary, search strategy and limitations, and AMSTAR items for *a priori* design, comprehensive literature search and list of studies provided to be adequate for most reviews. We noted the contrary however Cochrane reviews are consistently good at providing these items. We similarly noted AMSTAR items for publication bias and conflicts of interest to be poorly reported globally. McGee et al. did not perform any further statistical analyses to determine if there are any variables that predict higher review quality.

Braga et al. [180] evaluated the quality of systematic reviews and meta-analyses in paediatric urology published in major urological journals from 2000 to 2009 using the AMSTAR tool. 12 studies were included in the final analysis. They similarly identified poor reporting of the AMSTAR item 4 Inclusion of the grey literature. Contrary to our findings, they noted that *a priori* design, a full list of excluded studies and conflict of interests were provided by the majority of studies. We also identified a published conference abstract by Salim et al. [181] which evaluated the paediatric surgical literature using the AMSTAR tool. The authors appeared to have evaluated all systematic reviews in the field of paediatric surgery as opposed to those assessing surgical interventions alone as we did. 44 articles were included in their final analysis. Similar to our findings, publication bias is highlighted as a particularly poorly reported item with only 20% systematic reviews fulfilling this criteria adequately, and AMSTAR scores for items relating to duplicate study selection and comprehensive literature search being moderately well reported too.

### Weakness and limitations

Our review has of course its limitations. We attempted to identify all systematic reviews and meta-analyses since 2010 of surgical interventions in children in a pragmatic fashion as performed by a paediatric surgeon. This role itself is variable worldwide. Despite our best efforts, we may have missed articles either through the initial search or human error during the screening process. It is important to note that no MESH terms exist that are relevant to the specialties of paediatric surgery, paediatric urology or neonatal surgery. Ideally MESH terms would have been used in the initial search. Human error may also have affected the data extraction process. Furthermore, our scoring systems were binary in that AMSTAR and PRISMA criteria were either adequate or not, similar to the article by Adie et al. [12] It could be argued, however, that a scaled scoring system, such as that employed by McGee et al. [13], would have been more intuitive, accommodating for those criteria where adequacy was partly achieved. We minimised these limitations/risks by having two authors perform screening, selection and extraction independently, and interobserver reliability was high overall. We did not assess the grey literature, which may seem ironic considering our findings that systematic reviews and meta-analyses infrequently search this domain, but our aim was only to assess the published literature. It is also an assumption that if an AMSTAR or PRISMA item is not mentioned amongst the text of an manuscript that it did not occur. This, of course, will be false at times, although as mentioned earlier, no reviews which failed to mention registration were registered on PROSPERO. To our knowledge, neither PRISMA or AMSTAR scoring has not been formally validated. We are not aware of any research that has been published linking such scores with either effects of bias or an exaggeration of treatment effects. In our analysis, we allocated each article an aggregate score, however, this homogenises the quality assessment and is therefore a limitation of this study. By providing star charts (and the raw data), the reader may appreciate the adequacy of reporting of each AMSTAR and PRISMA criterion however. Finally, our secondary objective was to identify any article, author or journal characteristics associated with high quality reviews, however, we included all articles published within the period assessed and selected the exploratory characteristics to be used in regression modelling before yielding articles. Therefore, in total, we included 14 variables in regression analysis, some of which were inter-related, e.g. h-index and impact factor, or journal PRISMA endorsement and journal suggests PRISMA adherence. It would have been more statistically valid to limit the number of exploratory variables and avoid including closely associated variables.

We have highlighted areas for improvement in the literature, but we must consider means in which reporting and methodology of systematic reviews and meta-analyses in the surgery of childhood can be further improved. If more journals were to endorse PRISMA, or at least, to insist that authors adhere to its checklist, then the quality of reporting would be expected to improve. Of note, official and unofficial PRISMA endorsement were significant only on univariate linear regression, through articles mentioning PRISMA adherence was significantly associated with higher review quality in multiple regression analysis. Only the Cochrane Database of Systematic Reviews and PLOS ONE are official PRISMA endorsers, and only five other journals suggest adherence in their author guidelines, namely Annals of Surgery, BJU International, BMJ Open, the International Journal of Surgery, and the Journal of Trauma and Acute Care Surgery. Since more than half of all systematic reviews and meta-analyses in our study were published in the major paediatric surgical journals, their endorsement, or at least a change in their author guidelines, would have a significant impact in the quality of reporting in the specialty in the future.

## Conclusion

In conclusion, we have highlighted areas for improvement in quality of reporting and methodology of systematic reviews and meta-analyses in the paediatric surgical literature. *A priori* review registration, reviews including comparisons of interventions, and articles mentioning PRISMA, were characteristics associated with higher quality reviews. The latter variable is likely the reason why PRISMA adherence was not associated with higher review quality on final multivariate regression. Journals and investigators alike should take note of the benefits of PRISMA adherence in producing high quality systematic reviews and meta-analyses, which should have a positive impact on the accurate dissemination of knowledge to clinicians and in turn, the quality of surgical care received by children.

## Supporting information

S1 PRISMA Checklist PRISMA checklist items reported and their location within the text. (DOCX)

S1 Table The search strategy employed for EMBASE and MEDLINE. Last search performed on 10th June 2016.
(DOCX)

S2 Table Lists the hand-searched high impact journals. (DOCX)

S1 Dataset Raw cumulative AMSTAR and PRISMA scores for each systematic review analysed. Reporting.
(XLSX)
