# Supplementary material for: A systematic review of the quality of conduct and reporting of systematic reviews and meta-analyses in paediatric surgery
Source: PLoS One. 2017 Apr 6;12(4):e0175213. doi: 10.1371/journal.pone.0175213 (PMC5383307; doi:10.1371/journal.pone.0175213)
Supplement: S1 Table — Last search performed on 10th June 2016. (DOCX) [file pone.0175213.s002.docx]

| EMBASE, Medline |
| --- |
| (“PEDIATRIC SURGERY” OR “PAEDIATRIC SURGERY” OR “PEDIATRIC UROLOGY” OR “PAEDIATRIC UROLOGY” OR “SURGERY OF CHILDHOOD” OR “NEONATAL SURGERY”).af, combined with (“SYSTEMATIC REVIEW” OR “META-ANALYSIS” OR “QUANTITATIVE ANALYSIS” OR “DATA SYNTHESIS” OR “QUANTITATIVE OVERVIEW”) AND (PEDIATR* OR PAEDIATR* OR CHILD* OR INFANT* OR NEONAT* OR TODDLER OR ADOLESCENT OR JUVENILE).ti,ab [Limit to: publication year 2010-current]. |
