# Supplementary material for: A systematic review of the quality of conduct and reporting of systematic reviews and meta-analyses in paediatric surgery
Source: PLoS One. 2017 Apr 6;12(4):e0175213. doi: 10.1371/journal.pone.0175213 (PMC5383307; doi:10.1371/journal.pone.0175213)
Supplement: S2 Table — (DOCX) [file pone.0175213.s003.docx]

| **Journal type** | **Named journals searched** |
| --- | --- |
| General medical | New England Journal of Medicine  Lancet  BMJ  JAMA  PLOS ONE |
| Surgical | Annals of Surgery  British Journal of Surgery  Journal of the American College of Surgeons  JAMA Surgery  Journal of Trauma and Acute Care Surgery |
| Paediatrics | Pediatrics  JAMA Pediatrics  Journal of Pediatrics  Journal of Pediatric Gastroenterology and Nutrition  Archives of Disease of Childhood |
| Paediatric surgical | Journal of Pediatric Surgery  Pediatric Surgery international  European Journal of Paediatric Surgery  Journal of Pediatric Urology  Seminars in Pediatric Surgery |
